# Rare Cause of Pediatric Abdominal Pain Diagnosed on Point of Care Ultrasound (POCUS)

**DOI:** 10.24908/pocus.v9i2.17451

**Published:** 2024-11-15

**Authors:** Courtney Owens, Lindsey Chaudoin

**Affiliations:** 1 Atrium Health­Carolinas Medical Center Charlotte, NC USA

**Keywords:** Pediatric Abdominal Pain, OHVIRA Syndrome, Urogenital anomalies, POCUS Case Presentation

## Abstract

An 11-year-old postmenarchal female presented to the pediatric emergency department (ED) with 2 days of periumbilical and right lower abdominal pain. Radiology-performed ultrasound (RADUS) did not visualize the appendix, and there was a plan for surgical consultation to decide between serial abdominal exams versus computed tomography (CT) scan. While awaiting consultation and to help further narrow the differential diagnosis, the emergency provider performed a point of care ultrasound (POCUS) of the urinary tract. This revealed several anomalies including a solitary left kidney with hydronephrosis, and a well-circumscribed, fluid-filled structure with mixed echogenicity posterior to the bladder and inferior to the uterus. Given these findings on POCUS, further imaging was pursued, including a RADUS of the pelvis followed by a magnetic resonance imaging (MRI) of the abdomen. Further imaging ultimately diagnosed a bicornuate uterus, septate vagina with hematocolpos and solitary left kidney consistent with Obstructed Hemivagina and Ipsilateral Renal Anomaly (OHVIRA) syndrome. This case is an illustration of how POCUS is an invaluable tool to narrow the differential diagnosis and guide advanced imaging or consultation for both common and rare causes of pediatric abdominal pain.

## Case Presentation

An 11-year-old female with no known past medical history presented to the pediatric emergency department (ED) with 2-3 days of sharp abdominal pain. The pain was initially in the periumbilical region and then migrated to the right lower quadrant. There was no associated nausea, vomiting, diarrhea, constipation, vaginal discharge, vaginal bleeding, dysuria, hematuria, fevers or back pain. The patient’s menarche occurred at age 10, and her monthly menstrual cycles are without abdominal pain. The patient was first seen by her pediatrician and was referred to the ED for further evaluation.

Initial vital signs in the ED demonstrated a temperature of 37.2 C, blood pressure 128/66, pulse 126, respirations 18 and pulse oximetry of 100% on room air. Physical examination was notable for moderate tenderness to palpation of the periumbilical and right lower quadrant regions without rebound, guarding, mass or peritonitis, absent costovertebral angle tenderness, negative psoas sign and negative Rovsing’s sign. Initially, a broad differential diagnosis was considered, including appendicitis, ovarian torsion, urinary tract infection, nephrolithiasis and mesenteric lymphadenitis. Laboratory evaluation was remarkable for leukocytosis to 12,440 cells/µL (reference range 4.50-13.00 103/µL) with neutrophil predominance of 9,600 cells/µL (reference range 1.80-8.00 103/µL), and elevated CRP to 13.4 mg/dL (reference range <0.5 mg/dL). The complete metabolic panel and urinalysis were unremarkable, and her pregnancy test was negative.

**Figure 1:**
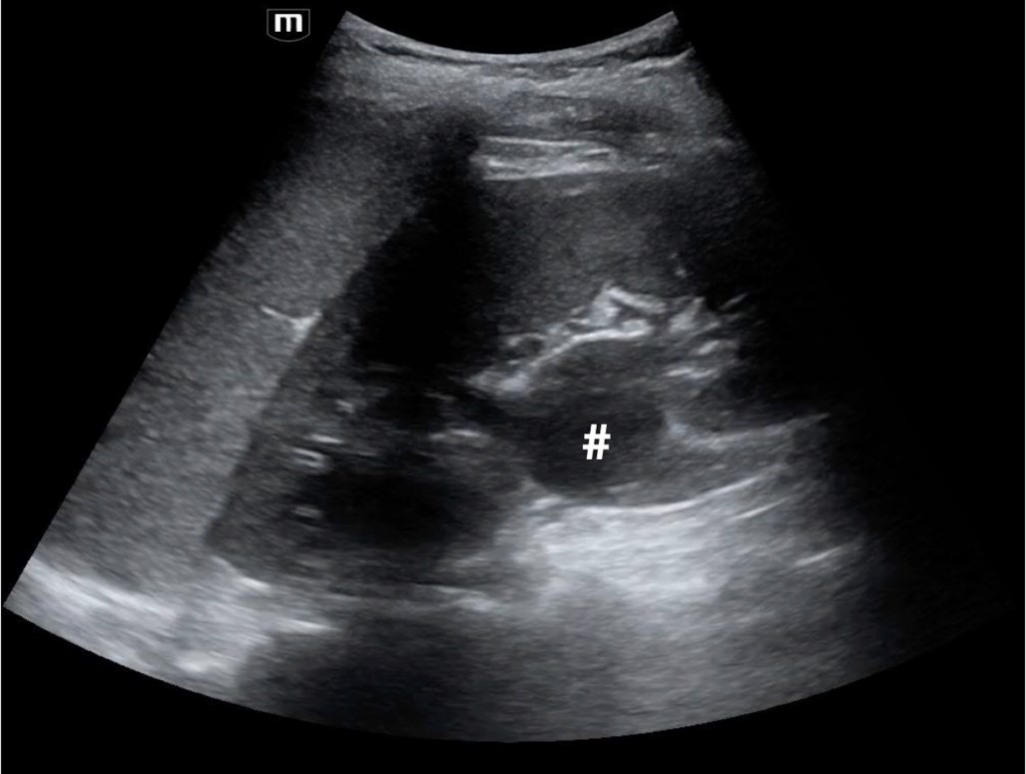
Left kidney with dilated renal pelvis (pound sign) representing mild hydronephrosis

**Figure 2:**
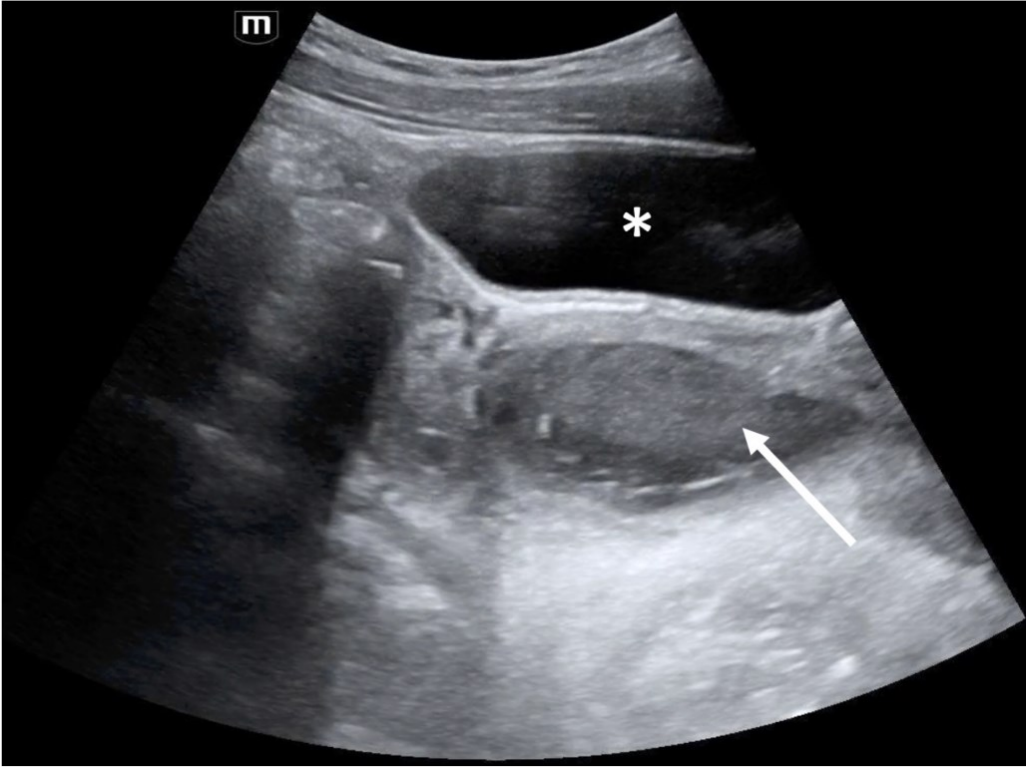
Pelvic view. The bladder was identified (asterisk), and posterior to the bladder was a well-circumscribed, complex fluid-filled area with mixed echogenicity (arrow)

**Figure 3:**
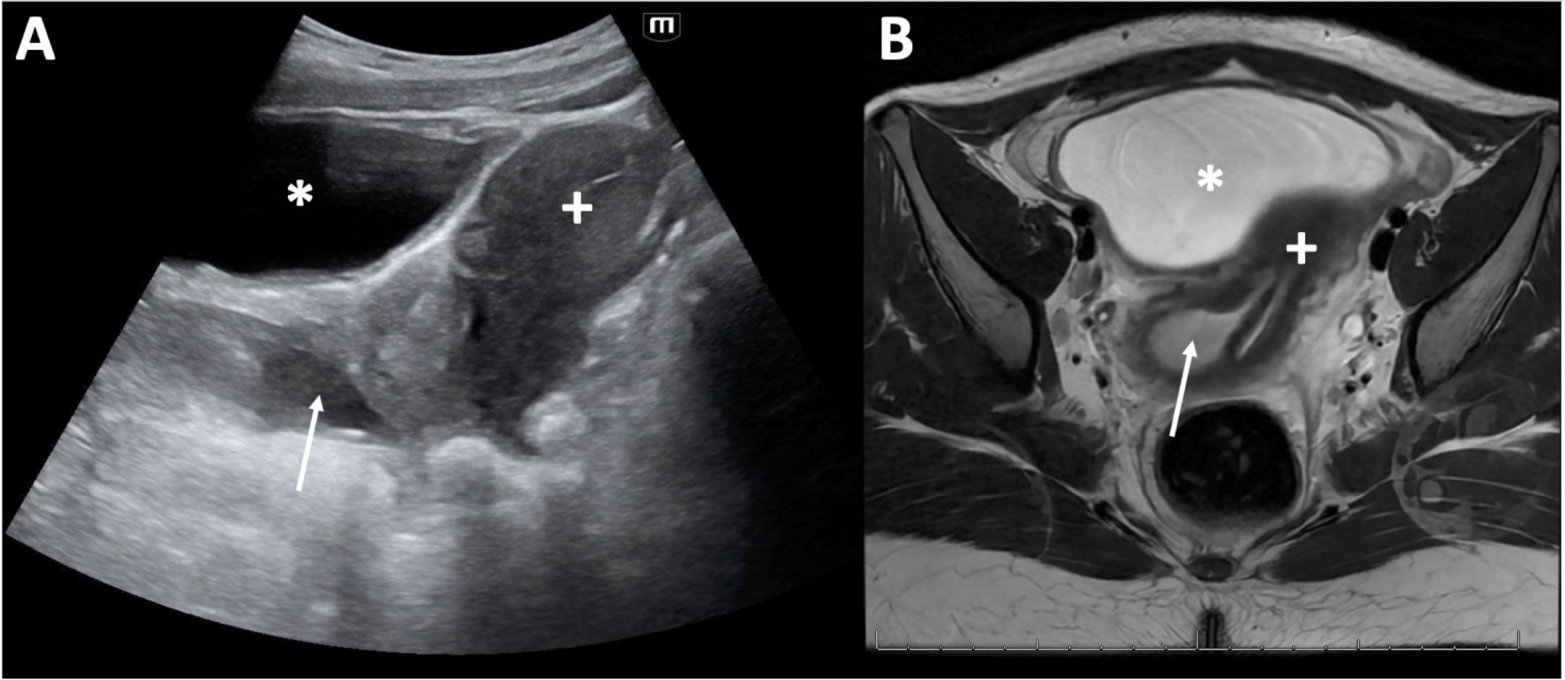
– Bladder (asterisk), uterus (plus sign), andhematocolpos in right side of septate vagina (arrow) initially visualized on point of care ultrasound (A) and redemonstrated on MRI (B).

There was heightened concern for appendicitis given right lower quadrant pain, leukocytosis, and elevated CRP. Radiology-performed ultrasound (RADUS) of the appendix was ordered, however, the appendix was not visualized. According to institutional protocols, pediatric surgery was consulted. While awaiting surgical consultation, the emergency provider performed a point of care ultrasound (POCUS) of the urinary tract to further narrow the differential diagnosis, and to specifically evaluate for hydronephrosis which would heighten concern for nephrolithiasis. The POCUS revealed a solitary left kidney with hydronephrosis (Figure 1) and a complex, fluid-filled structure posterior to the bladder (Figure 2). Given these abnormalities, pelvic POCUS was performed for further characterization which revealed that the complex, fluid filled structure appeared to be in either the vagina or rectum. RADUS of the pelvis and urinary tract was then ordered. The solitary left kidney was redemonstrated, and the pelvic ultrasound demonstrated a complete septate versus bicornuate uterus, and a longitudinal vaginal septum with hematocolpos of both canals, with right greater than left. The gynecology team was consulted and performed bedside decompression of the right hematocolpos, which relieved the patient’s pain. Gynecology recommended magnetic resonance imaging (MRI) for further characterization of pelvic anatomy with a plan to follow up with pediatric gynecology as an outpatient. MRI revealed a complete septate uterus with obstructing, longitudinal vaginal septum and moderately severe right hematocolpos, trace left pelvic free fluid, and right renal agenesis. MRI redemonstrated the right hematocolpos that was first visualized as a complex, fluid-filled structure posterior to the bladder on POCUS (Figure 3). On outpatient follow-up with pediatric gynecology, the patient was diagnosed with Obstructed Hemivagina and Ipsilateral Renal Anomaly (OHVIRA) syndrome. The patient was offered medical management with menstrual suppression versus surgical management with resection of hemivaginal septum. Patient opted for medical management with Depo-Provera and will follow up with pediatric gynecology every three months.

## Discussion

OHVIRA syndrome is a urogenital anomaly characterized by the presence of uterine didelphys, obstructed hemivagina and unilateral renal agenesis 1. OHVIRA syndrome, formerly known as Herlyn-Werner-Wunderlich syndrome, is exceedingly rare and thought to occur in less than 1 in 1,000,000 patients 1, 2. The exact etiology of OHVIRA syndrome is not completely understood, but is known to be the result of abnormal embryological development of the Wolffian and Mullerian ducts 3. In females, the Wolffian duct, or mesonephric duct, gives rise to the urinary tract system. Its abnormal development leads to the renal agenesis seen in OHVIRA syndrome 4, 5. The Wolffian duct is also responsible for inducing development of the Mullerian duct. The Mullerian duct, or paramesonephric duct, gives rise to the bilateral ovaries, uterus and upper third of the vagina. Abnormal Mullerian duct development leads to the uterine didelphys and obstructed hemivagina seen in OHVIRA syndrome 4, 5.

OHVIRA syndrome most commonly presents in adolescent, postmenarchal females with complaints of lower abdominal pain, dysmenorrhea or a tender vaginal mass 6. The presenting complaints are typically a result of the accumulation of menstrual blood in the obstructed hemivagina, known as hematocolpos. Given this, premenarchal diagnosis is rare and usually the result of antenatal ultrasound screening revealing unilateral renal agenesis 7. While most patients are diagnosed 1-2 years after menarche, the syndrome can go unrecognized, and misdiagnosed, well into adulthood due to normal menstruation through the unobstructed portion of the hemivagina 8.

Diagnosis of OHVIRA syndrome is often challenging due to the common and nonspecific presenting complaint of lower abdominal pain. Physical examination may show a vaginal bulge juxtaposed next to normal vaginal tissue due to hematocolpos, however, the frequency of this finding is unreported 9. The first-line diagnostic imaging modality is ultrasonography, which can reliably reveal renal agenesis and may reveal uterine didelphys, hematocolpos, and menstrual blood filling the obstructed hemivagina and uterus – known as hematometrocolpos 9. MRI is the gold standard diagnostic imaging modality which allows for complete visualization of urogenital anomalies and aids in intervention planning.

Management of OHVIRA syndrome is largely centered on correction of anatomic anomalies including drainage of hematocolpos, resection of vaginal septum, and – rarely – hemihysterectomy 10. Surgical management is usually recommended to prevent long term complications associated with retrograde menstruation, including development of tubal hematoma and abdominal endometriosis which can lead to eventual infertility 8, 10. Good prognosis of pregnancy outcomes are usually seen in patients who undergo appropriate intervention 8.

While the utility of POCUS in the diagnosis of OHVIRA syndrome is not well published, likely due to the rarity of the disease, it is evident that RADUS plays a critical role in identifying the urogenital anomalies that are diagnostic of the syndrome 1, 2, 6, 7, 8, 9. POCUS of the urinary tract as well as the nonpregnant female pelvis is within the scope of an emergency provider 11. While identification of complex abnormalities such as uterine didelphys or hematocolpos may be challenging for most emergency providers, absence of unilateral kidney is likely easily recognized and helpful in prompting further evaluation with additional diagnostic imaging as in this case and others in the literature 12.

This case demonstrates how POCUS was used to aid in the diagnosis of a rare pediatric urogenital anomaly, however, it also bears noting the utility of POCUS for identifying and treating other causes of pediatric abdominal pain. POCUS has been shown to be a valuable diagnostic tool in evaluating for prevalent pediatric abdominal pathologies, including appendicitis, pyloric stenosis, and ileocolic intussusception 13. Other abdominal and urogenital pathologies deemed to be within the scope of emergency provider performed POCUS for pediatric patients includes evaluating for traumatic intraabdominal free fluid, intrauterine pregnancy, testicular torsion, cholelithiasis, cholecystitis, and assessing bladder volume 14. In this case, POCUS was employed to evaluate the urinary tract for evidence of hydronephrosis which would raise concern for nephrolithiasis, a diagnosis that has traditionally been considered a less common cause of pediatric abdominal pain. Recent evidence demonstrates that the prevalence of pediatric nephrolithiasis is rising, specifically to have increased by 84.4% between 2006 to 2020 15. Beyond nephrolithiasis, hydronephrosis identified on POCUS can also raise concern for other causes of obstructive uropathy such as posterior urethral valves, phimosis, paraphimosis and constipation 16. Urinary tract POCUS has been shown to be a sensitive and specific method for detecting hydronephrosis within the adult population 17. However, there is a paucity of literature on the accuracy of pediatric urinary tract POCUS for assessing hydronephrosis 11, 18, 19. Further, to our knowledge, the few studies that do examine the use of pediatric urinary tract POCUS for hydronephrosis are all emergency provider performed studies on ED patients 18, 19. As the use of pediatric POCUS expands to various care spaces ranging from outpatient specialty clinics to the pediatric intensive care unit, it is evident that further future study is needed to determine the accuracy, utility, and feasibility of pediatric POCUS for the evaluation of both common, and previously thought rare, abdominal and urogenital pathologies 20.

## Conclusions

POCUS is an invaluable tool not only to narrow the differential diagnosis amongst more common causes of female pediatric abdominal pain, but also to help identify gross abnormalities that can prompt advanced imaging and consultation in patients with complex urogenital anomalies such as those found in OHVIRA syndrome. 

## Patient Consent and Disclosure

Parental consent for publication of this case was obtained by the authors. The authors declare that they have no competing interests.
